# New Synthetic Quinoline (Qui) Derivatives as Novel Antioxidants and Potential HSA’s Antioxidant Activity Modulators—Spectroscopic Studies

**DOI:** 10.3390/molecules28010320

**Published:** 2022-12-30

**Authors:** Wojciech Rogóż, Aleksandra Owczarzy, Karolina Kulig, Andrzej Zięba, Małgorzata Maciążek-Jurczyk

**Affiliations:** 1 Department of Physical Pharmacy, Faculty of Pharmaceutical Sciences in Sosnowiec, Medical University of Silesia, 40-055 Katowice, Poland; 2 Department of Organic Chemistry, Faculty of Pharmaceutical Sciences in Sosnowiec, Medical University of Silesia, 40-055 Katowice, Poland

**Keywords:** quinine derivatives, antioxidants, HSA, spectroscopy

## Abstract

The antioxidant activity of drugs, as well as the influence of drugs on the activity of endogenous antioxidant mechanisms in the human body is of great importance for the course of the disease and the treatment process. Due to the need to search for new therapeutic methods, the study of newly synthesized substances with potential therapeutic activity is necessary. This study aimed to designate some properties and characteristic parameters of new, synthetic quinoline three derivatives—1-methyl-3-allylthio-4-(4′-methylphenylamino)quinolinium bromide (Qui1), 1-methyl-3-allylthio-4-(3′-hydroxyphenylamino)quinolinium bromide (Qui2) as well as 1-methyl-3-allylthio-4-(4′-hydroxyphenylamino)quinolinium bromide (Qui3), including their antioxidant properties, as well as to analyse their activity as the potential modulators of Human Serum Albumin (HSA) antioxidant activity. In order to achieve the goal of the study, spectroscopic methods such as UV-Vis and circular dichroism (CD) spectroscopy have been used and based on the obtained data only slight and probably some surface interaction of quinoline derivatives (Qui1–Qui3) with HSA have been observed. The effect of Qui1–Qui3 on the HSA secondary structure was also insignificant. All analysed quinine derivatives have antioxidant activity against ABTS cation radical, in turn against DPPH radical, only Qui3 has noticeable antioxidant potential. The highest reduction potential by Qui3 as well as (Qui3 + HSA)_complex_ has been shown. Qui3 mixed with HSA has mostly the synergistic effect against DPPH, ABTS and FRAP, while Qui1 and Qui2 in the presence of HSA mostly have a synergistic and additive effect towards ABTS, respectively. Based on the obtained results it can be concluded that Qui2 and Qui3 can be considered potential modulators of HSA antioxidant activity.

## 1. Introduction

Many new medicinal substances are used to treat diseases and improve patients’ health. Quinine is an extremely important drug that has contributed to reducing the public health risk of malaria. From a “chemical point of view”, quinine is a natural derivative of quinoline (Qui). Additionally, synthetic quinoline derivatives are an extremely important group of chemical compounds, not only as therapeutic substances, but as models on the basis of which many conclusions can be drawn about their activity. Quinine is a naturally derived alkaloid that has been isolated from the bark of the *Cinchona* (*Rubiaceae*) tree [1,2]. This drug shows slightly analgesic effect and schizonticide activity (against presented in human erythrocytes parasites of the *Plasmodium* species), as well as gametocytocidal activity (against *Plasmodium vivax* and *P. malariae*). Currently, quinine is used to treat malaria in certain circumstances, for example in case of pregnant women with malaria, especially in the first trimester of pregnancy [3]. Although it is one of the best known antimalarial drug, it has been used less frequently in recent years due to the well-documented quinine side effects (cinchonism or quinine toxicity), including: acute bilateral blindness, headache, nausea, vomiting, visual symptoms and tinnitus [3,4,5,6]. Therefore, the searching for new substances with a potential therapeutic effect, including synthetic quinoline derivatives, is extremely important from the point of view of pharmaceutical and medical sciences. An important aspect of the activity of drugs used in infectious diseases, including those caused by parasites, is the possibility of the influence of free radicals on the human body.

Free radicals are molecules with unpaired electron on the valence shell. There are characterized by a very high chemical reactivity [7]. The high level of free radicals in human body makes damages to the patient’s health and leads to a longer recovery time [8]. For this reason living organisms have developed different ways of regulating the level of free radicals for example, enzymes (such as catalase), non-enzymatic antioxidants (such as some proteins) and methods of obtaining exogenous antioxidants (like vitamins). Both the antioxidant activity of individual drugs, as well as the possibility of modulation by them the endogenous antioxidant mechanisms significantly affect the course of the disease [7,8,9]. The possible effect of ligands-HSA interaction can be connected with the role of this protein in the processes of the human body’s free radicals regulating level [10]. HSA antioxidant activity is associated with the presence of free thiol group (-SH) in Cys-34 amino acid residue and, to a lesser extent, methionine (Met) residues [11,12,13,14]. The location of Cys-34 amino acid residue is in domain IA, at the HSA surface, close to Asp-38, His-39, and Tyr-84 residues and the availability of Cys-34 for ligands is limited due to the location of a side chain at the bottom of a crevice [15]. As Rogóż et al. showed, various drugs, such as losartan, furosemide, ketoprofen or naproxen, can modulate the antioxidant potential of HSA [10,16]. Quinoline (Qui) derivatives also influences on the ability of this protein to scavenge free radicals [17] and even slight chemical compound structure modifications can significantly change its properties. This, in turn, can contribute to improving as a modulator of the HSA antioxidant activity.

The primary goal of this study was to characterize the properties of three new and synthetic quinoline derivatives: 1-methyl-3-allylthio-4-(4′-methylphenylamino)quinolinium bromide (Qui1), 1-methyl-3-allylthio-4-(3′-hydroxyphenylamino)quinolinium bromide (Qui2) as well as 1-methyl-3-allylthio-4-(4′-hydroxyphenylamino)quinolinium bromide (Qui3), including their antioxidant properties and an examination of their interactions with HSA, as well as the potential in vitro modification of HSA antioxidant activity. A novel aspect of the present work was that none of the chemical compounds analyzed had ever been studied and described before. This means that all the results and conclusions described in this paper are novel and can significantly contribute to the development of science. Additional aims of the present study were also: to analyze the potential effects of the studied chemical compounds on the secondary structure of HSA (i); to investigate the possibility of obtaining effective modulators of antioxidant activity of HSA after the process of alkylation of known quinoline derivatives with allyl bromide (ii); to compare of the effects of different functional groups (methyl and phenyl groups) and their molecular distribution (meta and para position) of quinoline derivatives on their activity as antioxidants and modulators of antioxidant activity of HSA. Due to the very wide range of studies that are necessary to carry out, this work has been limited to spectroscopic methods.

## 2. Results and Discussion

### 2.1. Spectrophotometric (UV-Vis Spectroscopy) Analysis of the Qui1, Qui2, Qui3 and Their Interaction with HSA

**Figure 1:**
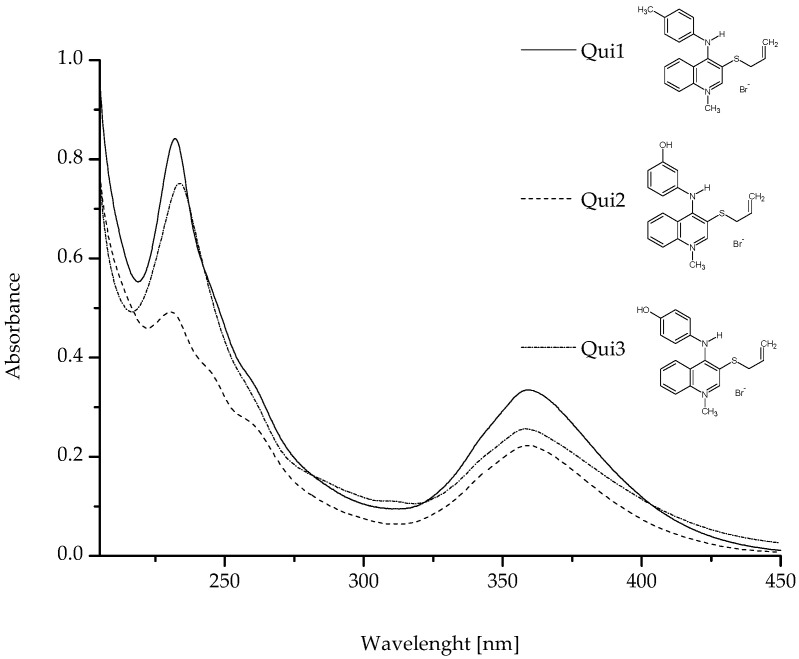
The absorption spectra of Qui1, Qui2 and Qui3 at 2.5 × 10^−5^ M concentration in phosphate buffer (pH = 7.4; 5 × 10^−2^ M).

In order to determine the properties of tested compounds (Qui1, Qui2 and Qui3), their spectroscopic analysis was performed. The absorption spectra of Qui1, Qui2 and Qui3 in phosphate buffer (pH = 7.4; 5 × 10^−2^ M) have been presented in Figure 1. The values of the samples’ mean molar absorptivity and mean absorbance have been collected in Table 1.

As can be observed (Figure 1), the spectroscopic spectra of the compounds Qui1, Qui2 and Qui3, especially the values of λ_max_ and λ_min_ as well as the absorbance, differ from each other. Moreover, the calculated molar absorptivity is not the same for Qui1–Qui3 samples (Table 1). This phenomenon means different abilities for radiation absorption, which is important for isomers Qui2 and Qui3. The only difference is that Qui2 contains a 3-hydroxyphenyl group while Qui3 contains a 4-hydroxyphenyl group. The hydroxyl group in Qui2 is in the meta position, and in Qui3 is in the para position. The differences in the absorption spectra between structural isomers of specific chemical compound were also analysed by Tsukamoto et al. [18]. They proved that ortho, meta and para isomers of (trifluoromethyl)benzoic acid are characterized by different absorption spectra in wavelength range between 190 nm and 300 nm and the meta isomer absorbance values at wavelengths from 215 nm to 240 nm were lower than for para isomer. Simultaneously the authors suggested, that the absorption band at 223 nm was a result of the presence of an aromatic ring in (trifluoromethyl)benzoic acid. Similarly in the present study the absorption band at wavelengths from 220 nm to 240 nm can be associated with the presence of methyl phenyl (Qui1) or hydroxyphenyl (Qui2, Qui3) groups (Table 1). The values of absorbance for Qui2 (meta isomer) were lower than for Qui3 (para isomer). This tendency is also specious for other structural isomers, such as benzenediols isomers: catechol, resorcinol and hydroquinone (ortho, meta and para isomers, respectively) and as Selvaraj et al. observed, the higher values of absorbance at wavelengths from 220 nm to 240 nm for hydroquinone than resorcinol has been obtained [19].

**Figure 2:**
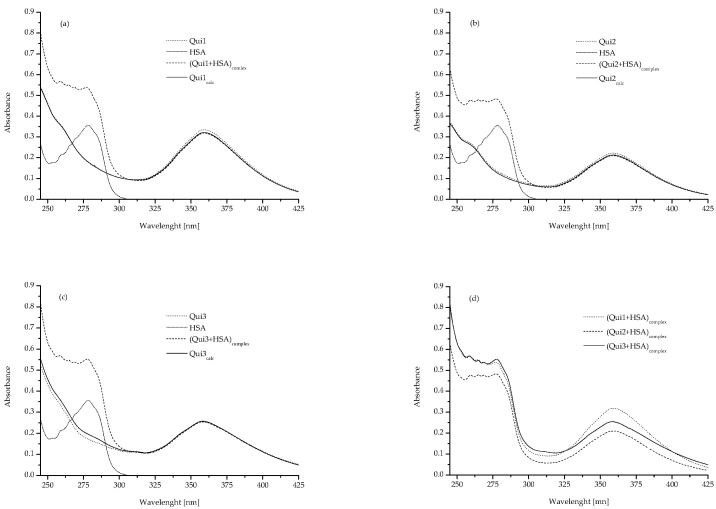
The absorption spectra of Qui1, HSA, (Qui1 + HSA)_complex_ and Qui1_calc_ = (Qui1 + HSA)_complex_ − HSA (**a**); Qui2, HSA, (Qui2 + HSA)_complex_ and Qui2_calc_ = (Qui2 + HSA)_complex_ − HSA (**b**); Qui3, HSA, (Qui3 + HSA)_complex_ and Qui3_calc_ = (Qui3 + HSA)_complex_ − HSA (**c**); (Qui1 + HSA)_complex_, (Qui2 + HSA)_complex_ and (Qui3 + HSA)_complex_ (**d**); Qui1, Qui2 and Qui3 concentration 5 × 10^−5^ M, HSA concentration 2.5 × 10^−5^ M; ligand: HSA molar ratio (in (Qui-HSA)_complex_) 2:1.

The examination of complex formation and the interaction of the mixture’s constituents can be done with the help of UV-Vis spectroscopy [20,21,22]. It is feasible to draw conclusion regarding the ligand-albumin interaction from the examination of the mixtures’ absorption spectra and those of their constituent parts (Figure 2).Figure 2 shows the absorption spectra of analysed samples and the absorbance mean values, both collected from Figure 2 (HSA, Qui1–Qui3, in the absence and presence of HSA) and mathematically calculated (Qui1_calc_, Qui2_calc_, Qui3_calc_), have been presented in Table 2.

Data presented in Table 2 allow us to determine the possibility of interaction between the components in the mixture. If the measured absorbance values of samples (Qui1–Qui3) are close to the mathematically calculated values of absorbance (Qui1_calc_–Qui3_calc_), it probably means that there is no interaction between the mixture components otherwise it may indicate the formation of ligand-protein complex [17,22]. Based on the spectra presented in Figure 2 no clear differences have been obtained, especially in case of Qui1 and Qui1_calc_ while the absorbance values for the analysed wavelengths differ both in the case of Qui2 and Qui2_calc_, as well as Qui3 and Qui3_calc_ samples (Table 2). Similar spectroscopic phenomena were carried out by Ren et al. They analysed the interaction mechanism between resveratrol and trypsin with the use of molecular docking and spectroscopic measurements. Using UV-Vis spectroscopy, the absorption spectra of trypsin, resveratrol, the mixture of resveratrol and trypsin, as well as the calculated spectrum of “(resveratrol + trypsin) − resveratrol” were determined. The absorption spectra of trypsin and the calculated spectrum of “(resveratrol + trypsin) − resveratrol” were slightly different. Based on this result they concluded, that both analysed substances formed a new complex [22]. Based on this observation it can be concluded that both Qui2 and Qui3 interact with HSA and complexes (Qui2 + HSA)_complex_ and (Qui3 + HSA)_complex_ are formed. Both of these chemical compounds (Qui2 and Qui 3) differ from Qui1 by the presence of a hydroxyl group. This may probably mean that the type of functional groups present in the ligand molecule influences its interactions with HSA (Figure 2, Table 2).

### 2.2. Spectropolarimetric Analysis of the Effect of Qui1, Qui2, Qui3 on HSA’s Secondary Structure

To investigate the influence of the tested substances on the secondary structure of HSA, measurements with the use of a CD spectropolarimeter have been performed. In Table 3 the values of the tested samples mean residue ellipticity [Θ_MRW_] have been collected.

Proteins are classified into two kinds based on the participation of individual secondary structural elements: α-helical proteins and β-type proteins. α-type proteins (dominant share of the α-helix structure) show two negative bands (two minima) in the CD spectrum at (λ_min_) ~209 nm and ~220 nm. In turn β-type proteins (dominant share of the β-sheet structure) are distinguished by having one, single negative band between λ 210 nm and 225 nm [23,24,25]. α_1_-acid glycoprotein (AGP), which represents β-sheet structure (percentage share is 83%), has a single negative band at λ 219.4 nm [26]. Taking into account literature data [26,27] and data presented above (two negative bands at 209 nm and 221 nm) it can be confirmed that regardless of ligand binding the dominant secondary structure of HSA is α-helix (Table 4). On the basis of the obtained CD spectra, the percentage content of individual components of the HSA’s secondary structure was determined and for this purpose Young’s reference model has been used.

Table 4 presents the percentage (%) content of the secondary structure elements of HSA, in the absence (HSA) and presence of ligands (Qui1–Qui3).

As it was shown in Table 4, small differences in the percentage (%) content of the secondary HSA structure have been recorded. Analysing of antibiotic agent (lomefloxacin) binding with HSA based on the CD, Beigoli et al. observed that the formation of the ligand-protein complex caused the reduction in the value of the percentage of HSA α-helix by ~5.6% as a result of certain degree of destabilization and conformational change of the protein [28]. Likewise, Matei et al. showed the change in α-helix content at the level of 2.3% when kaempferol interacted with HSA, calling this phenomenon “some degree of HSA defolding” [29]. Otherwise, as investigated by Owczarzy et al. the binding of 5-methyl-12(*H*)-quino[3,4-*b*][1,4]benzothiazinium chloride (Salt1) to HSA did not change significantly α-helix content (at a level of 0.5%) [26]. According to the data collected in Table 5, the presence of Qui1 in the solution did not a effect HSA’s secondary structure. Under Qui1 influence, the percentage share of α-helix and β-sheet have not changed. While in the case of Qui2 and Qui3, a slight changes in the percentage content of the α-helix structure of HSA’s secondary structure (decrease in the content of α-helix at the level of 1–2%) have been observed. Qui2 and Qui3 probably modified the HSA secondary structure, but this impact was very slight. Analysed samples showed some surface interaction with HSA (as it can be seen from the data in Figure 2, Table 2) but did not affect its conformation.

The preliminary spectroscopic characterization of the interaction between the studied ligands and HSA was an extensive introduction to the study of Qui1, Qui2 and Qui3 as potential modulators of HSA antioxidant activity. CD spectroscopy can be very helpful in studying changes in the antioxidant activity of albumin. Li et al. analysed the binding of α-tocopherol, ascorbic acid and proanthocyanidins with HSA and observed the changes in HSA’s secondary structure [30]. Similarly, Liu et al. analysed the interactions between quinoline alkaloids (quinine and quinidine) and BSA [31]. Based on the obtained CD spectra they showed that at a high concentration of quinine (5 × 10^−4^ M), there is a decrease in the α-helix percentage content in the secondary structure of BSA and it is probably related to the decrease in the content of hydrogen bonds in the HSA molecule [31]. The decrease in the number of highly structured structures most likely increases the accessibility of previously inaccessible locations, such as the free thiol group (-SH) in Cys-34 amino acid residue [14,15]. Quinine had a far stronger influence on BSA’s secondary structure than quinidine According to the Authors, the binding of quinine to BSA resulted in changes in the conformation of the protein, mainly due to the increased exposure of the hydrophobic regions of the protein and destabilization of hydrogen bonds [31]. It is noteworthy because this type of destabilization may result in a change in protein antioxidant activity probably due to the modified exposure of amino acid residues located on protein’s surface. The influence of quinine on HSA’s secondary structure has been also specified by Rogóż et al. [17]. They showed that the result of this reaction was an increase in α-helix content by ~1%. This allowed for the conclusion that analysed alkaloids slightly influenced HSA’s secondary structure.

Qui2 and Qui3 form complex with HSA and slightly change the percentage content of the α-helix (decrease in content of α-helix at the level of 1–2%). However, Qui1 derivative does not influence on both α-helix and β-sheet HSA content.

### 2.3. Spectrophotometric Analysis of the Antioxidant Activity of HSA, Qui1, Qui2 and Qui3 in Denaturing (DPPH Assay) and Non-Denaturing (ABTS Assay) Conditions

To characterize the investigated quinine derivatives and their influence on the properties of HSA, analysis with the use of model free radicals has been performed. Table 5 and Table 6 present the value of % inhibition and the value of AAEAC of Qui1–Qui3, HSA, (Qui1 + HSA)_complex_, (Qui2 + HSA)_complex_ and (Qui3 + HSA)_complex_ (DPPH assay, [Qui]:[HSA] 2:1 molar ratio), respectively.

DPPH assay is a very popular technique, usually one of the basic methods of detecting the antioxidant activity of newly synthesized compounds with potential therapeutic properties. 4-hydroxybenzoic acid (4-diethylamino-2-hydroxybenzylidene)hydrazide has been analysed by Sharma et al. [32]. They showed that the tested substance had antioxidant potential, but was lower than ascorbic acid. In turn, Geetha et al. [33] synthesized and analysed isoxazoline derivatives and on the basis of DPPH assay, a high level of differentiation of antioxidant activity of analysed samples they observed. Based on the data presented in Table 5 and Table 6 it can be concluded that both Qui2 and Qui3 showed antioxidant potential against DPPH model free radical. In contrast, Qui1 showed no DPPH radical scavenging ability. Because quinine dissolved in ethanol showed higher antioxidant activity than in the phosphate-buffered solution [17,34], the change of the solvent is suggested and it might cause higher antioxidant activity of Qui1 and Qui2 towards DPPH radical. Against the DPPH radical, the antioxidant potential of Qui1 and Qui2 oscillates close to zero while AAEAC values of Qui3 are higher. This allows the conclusion that Qui3 probably is a strong antioxidant (against DPPH radical). According to many authors, the location and type of functional group are important for antioxidant activity [35,36,37]. An increase in the number of hydroxyl groups usually increases antioxidant potential (highest antiradical potential: gallic acid > 3,4-dihydroksybenzoic acid > 4-hydroksybenzoic acid) [38]. The location of the hydroxyl group in the ortho (catechol), meta (resorcinol) and para (hydroquinone) positions (as well as other functional groups near them) are associated with an increase in antioxidant activity (highest antiradical potential: meta *<* para *<* ortho) [35,39]. This phenomenon confirms the accuracy of the observations presented in this work, because Qui3 (para isomer) is a stronger antioxidant than Qui2 (meta isomer).

DPPH assay contains denaturing conditions (the presence of ethanol). This means that the secondary and tertiary structure of the proteins analyzed by this technique is not native. Based on obtained results (Table 5 and Table 6) it can be concluded, that HSA (as well as HSA in the presence of Qui1–Qui3 in the environment) can retain its antioxidant properties despite changes in conformation. Various ligands, including antioxidants, drugs and radicals, can compete for binding sites on the surface of HSA. According to the observations of Li et al., the DPPH radical interacts with HSA, especially in the IIA subdomain where are also two classes of binding sites for DPPH [40]. They also said that one of the possible mechanisms behind the demonstration or even increase of the antioxidant activity of HSA in the mixture with ligands is the potential possibility of competition between radicals and ligands for the binding site on the surface of HSA. Based on the values presented in Table 6, AAEAC values obtained for ligand-HSA complexes were very different from each other. Regardless of the time that has elapsed since the initiation of the radical reaction (in the range of 5–60 min), the values of AAEAC for the (Qui3 + HSA)_complex_ were the highest. In turn, in the case of mixtures (Qui1 + HSA)_complex_ and (Qui2 + HSA)_complex_ much lower antioxidant activity has been shown. Qui1 and Qui2 differ from each other in terms of structure. Qui1 contains a methyl group in the para position, in turn Qui2 contains a hydroxyl group in the meta position. The presence of a hydroxyl group (Qui2) should be associated with higher antioxidant activity of the (Qui2 + HSA)_complex_. There was significant differences between the (Qui1 + HSA)_complex_ and (Qui2 + HSA)_complex_, because the antioxidant potential of the (Qui1 + HSA)_complex_ were higher than in case of (Qui2 + HSA)_complex_ (Table 6). This phenomenon may be probably due to the differences in the interaction of these two compounds with HSA. For example, based on the Zhang et al. [41] and Liu et al. [42] papers, it can be concluded that 9-hydroxyphenanthrene (9-OHPhe) and 9-methylphenanthrene (9-MP) differ only in one substituent (hydroxyl and methyl groups, respectively). Both compounds had a similar affinity for HSA, moreover reduction of α-helix content in the secondary structure of HSA and the ligand binding reaction with HSA were spontaneous. In turn, the complex created by HSA and 9-OHPhe was mainly stabilized by hydrophobic interactions and hydrogen bond forces (ΔH > 0; ΔS > 0) and in the case of the complex created by HSA and 9-MP van der Waals forces (ΔH < 0 ΔH < 0) have participated. The optimal binding site of ligands with HSA was also different. For 9-OHPhe it was the IA subdomain, in turn for 9-MP it was an environment between subdomains IB and IIA [41,42]. Based on the above data, it can be concluded that two very similar compounds can interact with HSA differently. To confirm the thesis that Qui1 and Qui2 react differently with HSA and thus have different effects on the HSA’s antioxidant activity, a calorimetric analysis of the binding of Qui1–Qui3 to HSA is necessary to do.

Expected and designated values of antioxidant activity AAEAC of the tested sample (DPPH assay) have been collected in Table 7.

Based on data collected in Table 7, it can be concluded that the result of the formation of the Qui3-HSA_complex_ is the occurrence of a synergistic effect in terms of HSA’s antioxidant potential after 5, 15, 25, and 45 min from the initiation of the radical reaction. Very importantly, in most cases expected and designated values of AAEAC were different (statistically significant). On this basis it is noteworthy that Qui3 probably is a strong HSA antioxidant activity modulator (Table 7). Similarly, there is possibility of modulating the antioxidant activity of HSA with quinine [17]. There are known cases of albumin ligands that the interaction between them and protein results in an antagonistic effect. For example, Yang et al. [44] with the use of a DPPH assay showed that the antioxidant activity of the mixture of curcumin and BSA is lower than expected (based on the results obtained for both components of the mixture separately). They drew similar conclusions from the analysis of BSA with the ι-carrageenan mixture [44]. Similarly, Wu et al. showed that the antioxidant activity of BSA and monascin mixture against DPPH radical is lower than the antioxidant activity of pure monascin solution [45]. Based on the above information it may be noted that in many cases ligands (also antioxidants) do not stimulate the albumin’s antioxidant activity.

For a comprehensive analyse the influence of Qui1–Qui3 on the HSA’s antioxidant activity, it is also necessary to perform the test under non-denaturing conditions (ABTS assay). Table 8 presents the values of AAEAC for Qui-Qui3, HSA, (Qui1 + HSA)_complex_, (Qui2 + HSA)_complex_ and (Qui3 + HSA)_complex_ ([Qui]:[HSA] 2:1 molar ratio), moreover Table 9 contains expected and designated values of antioxidant activity AAEAC of the tested samples.

The use of ABTS assay in antioxidant activity analyses in the case of newly synthesized chemical compounds was made by many authors [46,47]. Based on the data contained in Table 8 it can be concluded that Qui1–Qui3 showed antioxidant activity against the radical cation ABTS. The highest antioxidant potential of Qui2 has been shown. After 5 and 15 min from the initiation of the radical reaction the values of AAEAC in the case of Qui3 were higher than Qui1, while after 45 and 60 min the values of AAEAC in the case of Qui1 were higher than Qui3. There were large differences between the results obtained with DPPH and ABTS assays (Table 5, Table 6 and Table 8). All analysed quinine derivatives had antioxidant activity against ABTS cation radical (especially Qui2), in turn against DPPH radical only Qui3 had visible antioxidant potential. Obtained results are similar to results obtained by Aminjafari et al. [46]. They analysed the antioxidant potential of 4-aminocumarin derivatives. The sample marked as “comp 1” had the highest antioxidant activity against ABTS and DPPH. However, samples marked as “comp 2” and “comp 3” had no antioxidant activity against DPPH radical and notable antioxidant activity against ABTS (especially “comp 2”) [46]. Moreover, Naz et al. analysed the antioxidant activity of synthetic thioureas (compound I-V) with the use of DPPH and ABTS assays. Large differences between the results of both tests have been observed. The highest scavenging activities against DPPH radical had compounds IV and I, as well as against ABTS cation radical compound I and III. In both cases compound II was the weakest antioxidant, but compound IV also had very low antioxidant activity [47]. Significant differences in ABTS and DPPH assays results have been the subject of numerous analyses. This applies both to the analysis of food products [48] as well as animal tissue lysates [49]. Even small differences in the structure of antioxidants may affect their properties and activity against specific free radicals. The influence of the reaction environment and the chemical nature of the analysed antioxidant is also important. The above conclusions from the literature data correlate with the results of the present study. The hydroxyl group (presence and location) matters a lot for the antioxidant activity of chemical compound. The recorded differences can be explained by the absence of a hydroxyl group in Qui1 as well as differences in its localization in compounds Qui2 and Qui3. Although the use of both tests (DPPH and ABTS assays) allows for a comprehensive evaluation of the drugs, the ABTS assay shows a higher sensitivity, which can be seen both in this study (Table 6 and Table 8) and in the literature data [49].

On the other hand, AAEAC values obtained for ligand-HSA complexes were slightly different from each other. Regardless of the time that has elapsed since the initiation of the radical reaction (in the range of 5–60 min), the values of AAEAC for (Qui2 + HSA)_complex_ were the highest while AAEAC values of mixtures (Qui1 + HSA)_complex_ and (Qui3 + HSA)_complex_ were slightly lower than Qui2-HSA_complex_.

There are many possible effects of albumin interacting with ligands. For example, as Aime et al. has shown, Trolox (a strong antioxidant) in the presence of BSA (Bovine Serum Albumin) had pro-oxidative activity against N-(4-hydroxyphenyl)-trifluoroacetamide (CF(3)PAF). In turn, the pro-oxidative effect was not observed when urate, propyl gallate, glucose and carnosine were included in the reaction environment along with BSA [50]. On this basis it can be concluded that various ligands may not only enhance but also reduce albumin’s antioxidant activity. It may be related both to the possibility of blocking areas important from the point of view of radical reactions on the protein surface and to the influence of the course of the radical reaction by the ligand itself.

Based on the data collected in Table 9, it can be proposed that the result of the formation of the (Qui1 + HSA)_complex_ and (Qui3 + HSA)_complex_ is the occurrence of a synergistic effect in terms of HSA’s antioxidant potential. Moreover, in the case of the (Qui2 + HSA)_complex_ the additive effect can be observed. As Ihara et al. have demonstrated, an additive interaction effect between antioxidants and HSA is possible. Using ABTS assay, they showed that the mixture of lipophilic antioxidants (bilirubin or α-Tocopherol) and the antioxidant potential of HSA was very similar to the predicted one (based on the analysis of the antioxidant activity of their separate solutions). An additive effect of antioxidant-HSA interaction was also observed when hydrophilic antioxidant (ascorbic acid or uric acid) was used as ligand at some concentrations [51]. The observations of Ihara et al. are consistent with the presented in this work conclusions. Qui2 showed high antioxidant activity against ABTS cationic radical, and an additive interaction effect was observed in the case of the (Qui2 + HSA)_complex_ mixture. It can be concluded that all analysed quinine derivatives (Qui1–Qui3) probably are strong HSA’s antioxidant activity modulators in non-denaturing conditions (native HSA), against ABTS cation radical.

FRAP assay has been used to investigate the reduction potential of analysed samples. In Table 10 and Table 11 the values of ΔA and AAERP, respectively of Qui1–Qui3, HSA, (Qui1 + HSA)_complex_, (Qui2 + HSA)_complex_, (Qui3 + HSA)_complex_ (FRAP assay) have been collected.

Based on data presented in Table 10 and Table 11, it can be concluded that the highest reduction potential by Qui3 has been shown. In turn, in the case of Qui1 and Qui2 reduction potentials were very slight (especially in the case of Qui1). A very similar analysis of the reduction potential of synthetic analogy of 4-aminocoumarin (using FRAP assay) was carried out by Aminjafari et al. [46]. Their reference system was vitamin C. All tested compounds showed a very similar reduction potential to each other. This allowed for the conclusion that structural changes of molecules of the tested compounds were not associated with a change in the reduction potential [46]. In the present study, significant differences between the reduction potentials of the investigated quinoline derivatives were observed. This, in turn, allows for the conclusion that probably the hydroxylation of the phenolic group in the Qui3 para position is associated with its high reduction potential. The above statement can be confirmed by the Lu et al. studies [37]. They analysed the reduction potential of tocopherol derivatives with the use of FRAP assay, such as: (E)-Resveratrol-3-O-b-d-glucuronide (3-GR) and (E)-Resveratrol-4′-O-b-d-glucuronide (4′-GR). 3-GR had retained the 4′-OH group in the para position while 4′-GR is characterized by free hydroxyl group in meta position. As the Authors showed, 3-GR had higher reduction potential than 4′-GR and it probably means that the presence of the –OH group in the para position is very important for the compound’s reduction activity (like in the case of Qui3 in the present study) (Table 11). In turn 4′-GR showed a slight, but noticeable, reducing activity, similar to Qui2 (hydroxyl group in meta position; Table 11) [37].

Regardless of the time that has elapsed since the initiation of the radical reaction (in the range of 15–90 min), the values of ΔA and AAERP for Qui3-HSA_complex_ were the highest. Other Authors also used FRAP assay to study the reducing activity of the mixtures of the bioactive compounds [52]. From the various varieties of kiwi fruits, the highest reduction activity of *A. eriantha* cv. Bidan (“BC”) has been shown. “BC” was the mixture with the highest content of total phenolic content (TFC) and total ascorbic acid content (TAAC). In turn “AM” sample (*A. arguta* Cheongsan) had lower reduction potential, but this sample contained the highest level of total flavonoid content (TFC), total flavonoids content (TFL) and condensed tannin content (CTC) [52]. The cited work allowed to conclude that the reduction potential of the mixture might depend not only on the total composition of the mixture, but also on its individual components.

In the next step, the effects of Qui-protein complex formation were analysed (based on data obtained from Table 12) and results of analysis concerning the type of interaction between Qui and HSA have been shown (Table 12). Assuming that the result of Qui-protein complex formation is only an additive effect, the AAERP values obtained for both ligand (like Qui1) and mixture (like (Qui1 + HSA)_complex_) should be identical.

The FRAP assay is often used for the comparison of expected and designated (experimental and theoretical) results of antioxidant activity. Kopjar et al. analysed the ability of phenolics (catechin (C), quercetin (Q) and gallic acid (G)) as well as their mixtures to scavenge free radicals in the absence and presence of sugars (sucrose, trehalose), with the use of DPPH, ABTS and FRAP assays [53]. Using FRAP assay they showed, that the highest and the lowest antioxidant activity had the catechin and quercetin, respectively. In the absence of sugar only for catechin and quercetin mixture (CQ) the experimental value of the FRAP assay was lower than the theoretical one. The synergistic effect of interaction between phenolics in samples containing quercetin and gallic acid mixture (QG), catechin and gallic acid mixture as well as all analysed phenolics mixture (CQG) have been observed. Simultaneously Noguer at al. showed, that a synergistic effect of interaction is possible between uric acid and ascorbic acid (with the use of FRAP assay) [54]. Based on the above results it can be concluded, that various antioxidants can show different interaction effects. The synergistic effect of interaction is possible both between strong antioxidants and between antioxidants with lower antiradical activity. As shown in Table 13, only the formation of (Qui3 + HSA)_complex_ made it possible to obtain a synergistic effect of interaction (in the area of reduction potential) between quinine derivate and HSA (after 45, 60 and 90 min of incubation). For other samples the effect accompanying the preparation of sample mixtures was antagonistic. The main reason for the observed phenomenon was probably both the high reduction potential of Qui3 and the high activity of HSA. For the analysed concentration (2.5 × 10^−4^ M) of HSA no reduction potential has been observed (Table 10 and Table 11), but at other conditions, the presence of this activity is possible [55]. Based on this observation it may be concluded that Qui3 can stimulate the reduction potential of HSA and can be treated as HSA’s reduction activity modulator.

All analysed quinine derivatives had antioxidant activity against ABTS cation radical (especially Qui2), while against DPPH radical only Qui3 had antioxidant potential. The result of the formation of (Qui1 + HSA)_complex_ and (Qui3 + HSA)_complex_ is the occurrence of a synergistic effect in terms of HSA’s antioxidant potential. In turn, in case of (Qui2 + HSA)_complex_ the additive effect can be observed. The highest reduction potential by Qui3, both in the absence and presence of HSA ((Qui3 + HSA)_complex_) has been shown.

## 3. Materials and Methods

### 3.1. Chemicals

**Figure 3:**
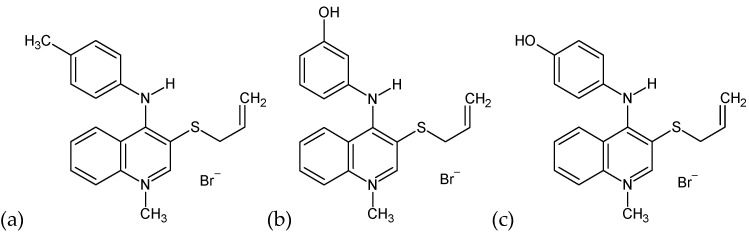
Structural formula of Qui1 (**a**), Qui2 (**b**) and Qui3 (**c**) (ChemSketch 12.1.0.31258).

Human serum albumin (HSA) was purchased from MP Biomedicals. 1-methyl-3-allylthio-4-(4′-methylphenylamino)quinolinium bromide (Qui1, Figure 3a), 1-methyl-3-allylthio-4-(3′-hydroxyphenylamino)quinolinium bromide (Qui2, Figure 3b) as well as 1-methyl-3-allylthio-4-(4′-hydroxyphenylamino)quinolinium bromide (Qui3, Figure 3c) were obtained by alkylating the corresponding 1-methyl-4-(phenylamino)quinolinio-3-thiolates (1-methyl-4-(4′-methylphenylamino) quinolinium-3-thiolate, 1-methyl-4-(3′-hydroksyphenylamino)quinolinium-3-thiolate, 1-methyl-4-(4′-hydroksyphenylamino)quinolinium-3-thiolate) with allyl bromide using the previously described method [2,56,57]. Spectroscopic parameters of the obtained compounds are given in Supplementary Materials. 2,2-Diphenyl-1-picrylhydrazyl (DPPH), 6-hydroxy-2,5,7,8-tetramethylchroman-2-carboxylic acid (TPTZ) and 2,2′-Azino-bis(3-ethylbenzothiazoline-6-sulfonic acid) diammonium salt (ABTS) were obtained from Sigma Aldrich. Potassium persulfate (PPS), hydrochloric acid (HCl) as well as ascorbic acid (AA) were purchased from Chempur. All chemicals were of analytical grade and used without further purification.

### 3.2. Methods

#### 3.2.1. Circular Dichroism (CD) Spectroscopy

All analysed samples have been measured using JASCO J-1500 Spectropolarimeter (JASCO International Co., Ltd., Hachioji, Tokyo, Japan). All solutions were prepared in phosphate buffer (pH = 7.4; 5 × 10^−2^ M) at room temperature. The absorption spectra of the samples were analysed in the wavelength range from 200 to 250 nm with 1 mm path-length quartz cuvettes. A thermostatic Peltier cell holder, with an accuracy of ±0.05 °C, has been used. The stock concentration of Qui1–Qui3 ([Qui1] = [Qui2] = [Qui3]) was 3 × 10^−4^ M, HSA concentration was 3 × 10^−6^ M as well as in the complex with Qui1–Qui3 6 × 10^−6^ M ([Qui1–Qui3]:[HSA] 2:1 molar ratios).

The mean residue ellipticity [Θ]_MRW_ was calculated using Equation (1) [58,59]:

(1) $$\left[\Theta {]}_{\mathrm{MRW}}=\frac{\mathrm{MRW}\times \Theta }{10\times l\times m}\;[\deg\times {\mathrm{cm}}^{2}\times {\mathrm{dmol}}^{-1}\right]$$

where:

Θ—observed ellipticity for a given wavelength [deg]; m—the protein concentration [g × cm^−3^]; l—the pathlength [cm]; MRW—a mean residue weight (MRW_HSA_ = 113.7 Da).

#### 3.2.2. UV–Vis Spectroscopy

The samples have been measured using JASCO V-730 UV-Visible Spectrophotometer (JASCO International Co., Ltd., Hachioji, Tokyo, Japan). All sample solutions were prepared in phosphate buffer (pH = 7.4; 5 × 10^−2^ M) and tested at room temperature.

##### Antioxidant Activity

DPPH (2,2-difenylo-1-pikrylohydrazyl radical), ABTS (2,2′-azino-bis(3-ethylbenzothiazoline-6-sulfonic acid diammonium salt) and FRAP (mixture of TPTZ (2,4,6-Tri(2-pyridyl)-s-triazine) in hydrochloric acid), assays were prepared based on the Rogóż et al. protocol [10,16].

In the DPPH assay ethanolic solution of DPPH at 1 × 10^−4^ M concentration as a working reagent has been used. The concentration of Qui1–Qui3 ([Qui1] = [Qui2] = [Qui3]) was 2 × 10^−4^ M, HSA concentration was 1 × 10^−4^ M, as well as in the complex with Qui1–Qui3 (1 × 10^−4^ M) was 5 × 10^−5^ M ([Qui1–Qui3]:[HSA] 2:1 molar ratios). Samples of Qui1–Qui3, HSA, and Qui1–Qui3 + HSA solutions were mixed with DPPH in the volume ratio (*v/v*) 1:1. The absorbance measurements were performed for the maximum absorption of DPPH at 517 nm, after 5, 15, 25, 45 and 60 min from the moment of initiation of the radical reaction between DPPH and tested sample. During the radical reaction all tested samples were stored in dark at room temperature.

To perform the ABTS assay, a working reagent was prepared. ABTS after a reaction with potassium persulfate can be converted to cationic radical. The concentration of Qui1–Qui3 ([Qui1] = [Qui2] = [Qui3]) was 8 × 10^−6^ M, HSA concentration was 4 × 10^−6^ M, as well as in the complex with Qui1–Qui3 (at 4 × 10^−6^ M concentration) was 2 × 10^−6^ M ([Qui1–Qui3]:[HSA] 2:1 molar ratios). Samples of Qui1–Qui3, HSA, and Qui1–Qui3 + HSA solutions were mixed with ABTS cationic radical solution in the volume ratio (*v/v*) 1:1. The absorbance measurements were performed for the maximum absorption of ABTS at 734 nm, after 5, 15, 30, 45 and 60 min from the moment of initiation of the radical reaction between ABTS and tested sample. During the radical reaction all tested samples were stored in dark at room temperature.

The % inhibition value has been calculated based on the following Equation (2) [60,61]:

(2) $$\%\;\mathrm{inhibition}=\left(\frac{A_{0}-A_{1}}{A_{0}}\right)\times 100\%$$

where:

A_0_, A_1_—the initial absorbance of DPPH or ABTS, in the absence and presence of the samples, respectively.

In the FRAP assay as a working reagent (FRAP reagent) mixture of TPTZ at 1 × 10^−2^ M concentration with hydrochloric acid (4 × 10^−2^ M), a water solution of iron chloride (III) (2 × 10^−2^ M) and acetate buffer (at pH 3.6) in volume ratio 1:1:10 have been used. The protocol was prepared based on Benzie et al. work [55]. The concentration of Qui1–Qui3 ([Qui1] = [Qui2] = [Qui3]) was 5 × 10^−4^ M, HSA concentration was 2.5 × 10^−4^ M ([Qui1–Qui3]:[HSA] 2:1 molar ratios). Samples of Qui1–Qui3, HSA, Qui1–Qui3 + HSA solutions were mixed with FRAP reagent in the volume ratio (*v/v*) 1:7.5. The absorbance measurements were performed for the maximum absorption of FRAP at 593 nm, after 15, 30, 45, 60 and 90 min from the moment of initiation of the reaction between FRAP and tested sample. During the radical reaction all tested samples were stored in dark at 310 K temperature.

The obtained results in FRAP assay as an ΔA value have been shown. The ΔA value has been calculated based on the following Equation (3):

ΔA = A_1*_ − A_0*_ (3)

where:

A_0*_, A_1*_—the initial absorbance of FRAP reagent, in the presence of phosphate buffer (pH = 7.4; 5 × 10^−2^ M), without and in the presence of the samples, respectively.

To compare the ability of the tested samples to scavenge free radicals with ascorbic acid (AA), calibration curves were prepared (Table 13). The relationship between the concentration of AA and the value of % inhibition or ΔA for these concentrations has been determined. Calibration curve patterns as well as R^2^ (for DPPH, ABTS and FRAP assays) were presented in Table 13.

Based on the calibration curves (Table 13), % inhibition or ΔA values of the tested samples were converted into the concentration of ascorbic acid (AA), which is reflected in AAEAC (Ascorbic Acid Equivalent Antioxidant Capacity; DPPH, ABTS assays) or in AAERP (Ascorbic Acid Equivalent Reduction Potential; FRAP assay).

To analyse the type of interaction between HSA and ligands, the values of expected (“Ex”) AAEAC of the tested samples were determined. The procedure for interaction type analysis was prepared based on the work of Guimarães et al. [43]. The “Ex” values of AAEAC were determined as a weighted average of the AAEAC values obtained for the components of the mixture (HSA and Qui1–Qui3) separate and in the mixture.

##### Quinine Derivatives—HSA Interaction

The absorption spectra of Qui1, Qui2 and Qui3 solutions, without and in the presence of HSA (2.5 × 10^−5^ M) were analysed when the concentration was 2.5 × 10^−5^ M and 5 × 10^−5^ M, respectively, the wavelength range was from 210 to 450 nm with 10 mm path length quartz cuvettes.

The obtained results of molar absorptivity analysis have been calculated with the use of Equation (4) [62]:

A = ɛ × c × l(4)

where:

A—samples absorbance, ɛ—molar absorptivity [dm^3^ × mol^−1^ × cm^−1^], c—samples concentration [mol × dm^−3^], l—optical path length (10 mm).

Ligand-protein mixtures were prepared in volume ratio (*v/v*) 1:1 (ligand: HSA molar ratio (in Qui-HSA_complex_)] 2:1). All the spectroscopic spectra of HSA were subtracted from (Qui1 + HSA)_complex_, (Qui2 + HSA)_complex_ and (Qui3 + HSA)_complex_, giving spectra of Qui1, Qui2 and Qui3. For the interaction analysis, similarly as in the protocol described by Rogóż et al. [16], to obtain Qui1_calc_, Qui2_calc_ and Qui3_calc_, the mathematical calculation (Qui_calc_ = (Qui + HSA)_complex_ minus HSA) have been performed.

### 3.3. Statistics

All results of the analysed samples were repeated minimum in triplicate and expressed as a mean ± relative standard deviation (mean ± SD). The obtained results were analysed using OriginPro software version 8.5 SR1 (Northampton, MA, USA), Statistica (data analysis software system), version 13; TIBCO Software Inc. 2017 (Palo Alto, CA, USA), Microsoft Excel 2013 (Redmond, WA, USA) and Spectra Manager Version 2.13.00 2002–2015 (JASCO International CO., LTD., Hachioji, Tokyo, Japan).

## 4. Conclusions

Qui1–Qui3 are newly synthesized quinine derivatives. The present work allowed to realize the main aim, which was to determine the antioxidant properties of Qui1–Qui3, as well as their activity as potential modulators of the antioxidant activity of human serum albumin (HSA).

Based on the conducted studies it was possible to conclude that the tested compounds, although similar to each other, were characterized by a variety of properties. All compounds tested had an influence on the antioxidant activity of HSA. Qui3 was probably the most effective modulator of HSA antioxidant activity. Based on the performed analyses, it can be concluded that the process of alkylation of known quinoline derivatives with allyl bromide may allow obtaining compounds affecting the free radical scavenging ability of HSA. The presence of a hydroxyl group in the molecule of the newly synthesized compound and its location in the para position could mean that the substance probably interacts with HSA and positively affect its antioxidant potential. Spectroscopic methods are an effective method not only for detecting the properties of newly synthesized chemical compounds, but also allow inferences about their potential toxicity.

Based on obtained results it can be concluded that among the tested compounds Qui2 and Qui3 showed antioxidant activity and can be considered potential modulators of the antioxidant activity of HSA. Further studies are needed to further characterize not only Qui1–Qui3 compounds, but also other quinoline derivatives.

## Figures and Tables

**Table 1:** The values of Qui1, Qui2 and Qui3 mean molar absorptivity and mean absorbance at 2.5 × 10^−5^ M concentration in phosphate buffer (pH = 7.4; 5 × 10^−2^ M).

Sample	Wavelength λ [nm]	Absorbance (Mean ± SD *)	Molar Absorptivity (ɛ) [dm^3^ × mol^−1^ × cm^−1^] (Mean ± SD *)
Qui1	λ_max1_ 232.0	0.842 ± 0.002	33677 ± 65
	λ_max2_ 358.8	0.335 ± 0.001	13387 ± 45
	λ_min1_ 218.8	0.553 ± 0.002	22105 ± 68
	λ_min2_ 312.6	0.094 ± 0.001	3773 ± 49
Qui2	λ_max1_ 230.4	0.492 ± 0.000	19682 ± 20
	λ_max2_ 358.6	0.222 ± 0.001	8876 ± 32
	λ_min1_ 222.2	0.459 ± 0.001	18380 ± 29
	λ_min2_ 312.6	0.064 ± 0.001	2541 ± 17
Qui3	λ_max1_ 233.6	0.752 ± 0.000	30088 ± 16
	λ_max2_ 358.2	0.256 ± 0.000	10249 ± 18
	λ_min1_ 216.2	0.492 ± 0.001	19662 ± 21
	λ_min2_ 318.0	0.105 ± 0.001	4209 ± 28

* Standard deviation.

**Table 2:** The values of mean absorbance, both experimentally measured and mathematically calculated of Qui1, Qui2, Qui3, as well as Qui1_calc_ = (Qui1 + HSA)_complex_ − HSA, Qui2_calc_ = (Qui2 + HSA)_complex_ − HSA and Qui3_calc_ = (Qui3 + HSA)_complex_ − HSA; Qui1, Qui2 and Qui3 concentration 2.5 × 10^−5^ M, HSA concentration 1.25 × 10^−5^ M, ligand: HSA molar ratio (in (Qui-HSA)_complex_) 2:1.

Sample	Wavelength λ [nm]	Absorbance (Mean ± SD *)	
		Measured	Mathematically Calculated (Qui_calc_)
Qui1	250.8	0.449 ± 0.001	0.445 ± 0.001
	267.2	0.268 ± 0.001	0.268 ± 0.002
	278.6	0.177 ± 0.001	0.178 ± 0.000
	291.2	0.126 ± 0.001	0.126 ± 0.001
	358.8	0.335 ± 0.001	0.322 ± 0.001
Qui2	250.8	0.312 ± 0.000	0.307 ± 0.001
	263.8	0.240 ± 0.000	0.234 ± 0.001
	278.6	0.134 ± 0.001	0.124 ± 0.002
	285.0	0.111 ± 0.000	0.102 ± 0.003
	358.6	0.222 ± 0.001	0.213 ± 0.003
Qui3	250.8	0.421 ± 0.000	0.445 ± 0.042
	265.6	0.261 ± 0.000	0.284 ± 0.025
	278.6	0.172 ± 0.000	0.195 ± 0.017
	285.0	0.154 ± 0.000	0.173 ± 0.016
	358.2	0.256 ± 0.000	0.256 ± 0.021

* Standard deviation.

**Table 3:** The CD spectra and the values of the mean residue ellipticity [Θ_MRW_] of HSA, both in the absence (HSA) and presence of ligands ((Qui1 + HSA)_complex_, (Qui2 + HSA)_complex_ (Qui3 + HSA)_complex_) ([Qui]:[HSA] 2:1 molar ratio)).

** 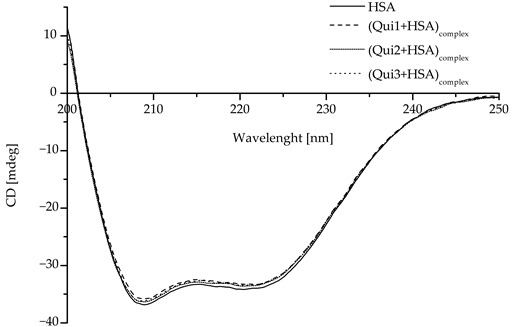 **		
**Sample**	**λ_min_[nm]**	**[Θ_MRW_]** **[deg × cm^2^ × dmol^−1^]** (Mean ± SD *)
HSA	209	−20,922 ± 65
	221	−19,371 ± 75
(Qui1 + HSA)_complex_	209	−20,459 ± 153
	221	−18,994 ± 82
(Qui2 + HSA)_complex_	209	−20,793 ± 146
	221	−19,141 ± 114
(Qui3 + HSA)_complex_	209	−20,533 ± 80
	221	−19,021 ± 8

* Standard deviation.

**Table 4:** The percentage (%) content of the secondary structure elements of HSA in the absence (HSA) and presence of ligands (Qui1–Qui3); Young’s reference model.

Sample	% α-Helix (Mean ± SD *)	% β-Sheet (Mean ± SD *)	% Turn (Mean ± SD *)	% Other (Mean ± SD *)
HSA	34.17 ± 0.21	13.43 ± 0.12	20.70 ± 0.17	31.73 ± 0.12
(Qui1 + HSA)_complex_	34.10 ± 0.69	13.77 ± 0.46	20.47 ± 0.06	31.70 ± 0.26
(Qui2 + HSA)_complex_	32.70 ± 0.10	13.77 ± 0.21	20.97 ± 0.15	32.57 ± 0.06
(Qui3 + HSA)_complex_	33.40 ± 0.10	13.37 ± 0.25	20.93 ± 0.21	32.23 ± 0.15

* Standard deviation.

**Table 5:** The value of % inhibition of Qui1–Qui3 ([Qui1] = [Qui2] = [Qui3] = 2 × 10^−4^ M), HSA ([HSA] = 1 × 10^−4^ M), and (Qui1 + HSA)_complex_, (Qui2 + HSA)_complex_, (Qui3 + HSA)_complex_ ([Qui] = 1 × 10^−4^ M; [HSA] = 5 × 10^−5^ M; [Qui]:[HSA] 2:1 molar ratio); DPPH assay.

DPPH Assay % Inhibition (Mean± SD *)					
Samples	Time [min]				
	5	15	25	45	60
HSA	4.48 ± 1.73	10.35 ± 5.07	14.07 ± 1.82	15.54 ± 3.84	20.45 ± 4.54
Qui1	ND **	0.00 ± 2.09	4.26 ± 3.71	3.39 ± 6.66	ND **
Qui2	ND **	2.90 ± 0.43	6.41 ± 1.27	5.00 ± 2.41	3.58 ± 0.05
Qui3	20.09 ± 1.44	17.94 ± 4.36	19.81 ± 3.63	22.69 ± 1.52	17.83 ± 1.99
(Qui1 + HSA)_complex_	7.99 ± 1.97	11.58 ± 4.62	15.59 ± 0.89	16.58 ± 3.10	12.87 ± 1.93
(Qui2 + HSA)_complex_	5.40 ± 0.25	7.02 ± 4.38	10.06 ± 0.33	10.03 ± 4.50	9.92 ± 8.44
(Qui3 + HSA)_complex_	25.08 ± 0.67	25.17 ± 2.24	21.56 ± 6.34	25.57 ± 0.20	19.25 ± 1.35

* Standard deviation. ** ND—No data; indeterminate values due to too low Qui1 and Qui2 antioxidant activity in DPPH assay.

**Table 6:** The Total Antioxidant Capacity (AAEAC); ([Qui1] = [Qui2] = [Qui3] = 2 × 10^−4^ M), HSA ([HSA] = 1 × 10^−4^ M), and (Qui1 + HSA)_complex_, (Qui2 + HSA)_complex_, (Qui3 + HSA)_complex_ ([Qui] = 1 × 10^−4^ M; [HSA] = 5 × 10^−5^ M; [Qui]:[HSA] 2:1 molar ratio); DPPH assay.

DPPH Assay Antioxidant Activity AAEAC (Mean ± SD *) [µM AA]					
Samples	Time [min]				
	5	15	25	45	60
HSA	2.25 ± 1.01	4.11 ± 3.10	5.95 ± 1.13	6.90 ± 2.47	9.54 ± 3.00
Qui1	ND **	ND **	ND **	ND **	ND **
Qui2	ND **	ND **	1.19 ± 0.79	0.12 ± 1.55	ND **
Qui3	11.40 ± 0.84	8.75 ± 2.66	9.51 ± 2.25	11.50 ± 0.98	7.81 ± 1.32
(Qui1 + HSA)_complex_	4.31 ± 1.15	4.86 ± 2.83	6.89 ± 0.55	7.56 ± 2.00	4.54 ± 1.27
(Qui2 + HSA)_complex_	2.79 ± 0.15	2.07 ± 2.68	3.46 ± 0.20	3.36 ± 2.89	2.59 ± 5.57
(Qui3 + HSA)_complex_	14.32 ± 0.39	13.18 ± 1.37	10.59 ± 3.94	13.35 ± 0.13	8. 75 ± 0.89

* Standard deviation. ** ND—No data; indeterminate values due to too low Qui1 and Qui2 antioxidant activity in DPPH assay.

**Table 7:** Expected (Ex) versus designated (De) values of antioxidant activity AAEAC of the (Qui3 + HSA)_complex_; DPPH assay.

DPPH Assay Antioxidant Activity AAEAC (Mean ± SD *) [µM AA]						
Sample		Time [min]				
		5	15	25	45	60
(Qui3 + HSA)_complex_	De	14.32 ± 0.39	13.18 ± 1.37	10.59 ± 3.94	13.35 ± 0.13	8.75 ± 0.89
	Ex	6.82 ± 0.49	6.43 ± 1.84	7.73 ± 1.48	9.20 ± 0.75	8.68 ± 0.84
	Effect	s ^#^	s ^#^	s	s ^#^	add

* Standard deviation. ^#^ Statistically significant difference between Ex and De. Ex—expected; De—designated; add—an additive effect: expected and designated values reveal differences lower than 5%. s—a synergistic effect: designated values are more than 5% lower for AAEAC when compared with expected values. an—an antagonistic effect: designated values are more than 5% higher for AAEAC when compared with expected values [43].

**Table 8:** The Total Antioxidant Capacity (AAEAC); ([Qui1] = [Qui2] = [Qui3] = 8 × 10^−6^ M), HSA ([HSA] = 4 × 10^−6^ M), and (Qui1 + HSA)_complex_, (Qui2 + HSA)_complex_, (Qui3 + HSA)_complex_ ([Qui] = 4 × 10^−6^ M; [HSA] = 2 × 10^−6^ M; [Qui]:[HSA] 2:1 molar ratio); ABTS assay.

ABTS Assay Antioxidant Activity AAEAC (Mean ± SD *) [µM AA]					
Samples	Time [min]				
	5	15	30	45	60
HSA	14.97 ± 0.63	25.63 ± 0.51	35.24 ± 0.88	40.96 ± 0.71	44.04 ± 1.22
Qui1	1.06 ± 0.67	9.23 ± 0.96	19.45 ± 0.78	24.48 ± 1.22	27.57 ± 1.20
Qui2	33.24 ± 1.59	35.47 ± 1.64	38.91 ± 1.75	41.16 ± 2.02	41.94 ± 1.76
Qui3	19.67 ± 4.12	20.70 ± 4.19	21.81 ± 2.81	21.91 ± 3.15	21.63 ± 2.69
(Qui1 + HSA)_complex_	9.30 ± 0.22	19.56 ± 0.18	29.51 ± 0.30	35.71 ± 0.48	40.58 ± 0.22
(Qui2 + HSA)_complex_	25.02 ± 0.35	30.25 ± 0.37	36.81 ± 0.84	41.52 ± 1.00	43.93 ± 0.81
(Qui3 + HSA)_complex_	21.06 ± 0.24	25.93 ± 0.06	32.81 ± 0.06	38.06 ± 0.47	40.69 ± 0.70

* Standard deviation

**Table 9:** Expected versus designated values of antioxidant activity AAEAC of the tested samples; ABTS assay.

ABTS Assay Antioxidant Activity AAEAC (Mean ± SD *) [µM AA]						
Samples		Time [min]				
		5	15	30	45	60
(Qui1 + HSA)_complex_	De	9.30 ± 0.22	19.56 ± 0.18	29.51 ± 0.30	35.71 ± 0.48	40.58 ± 0.22
	Ex	8.02 ± 0.65	17.43 ± 0.65	27.35 ± 0.76	32.72 ± 0.81	35.80 ± 1.10
	Effect	s ^#^	s ^#^	s ^#^	s ^#^	s ^#^
(Qui2 + HSA)_complex_	De	25.02 ± 0.35	30.25 ± 0.37	36.81 ± 0.84	41.52 ± 1.00	43.93 ± 0.81
	Ex	24.11 ± 0.50	30.55 ± 0.59	37.08 ± 0.46	41.06 ± 0.79	42.99 ± 0.49
	Effect	add ^#^	add	add	add	add
(Qui3 + HSA)_complex_	De	21.06 ± 0.24	25.93 ± 0.06	32.81 ± 0.06	38.06 ± 0.47	40.69 ± 0.70
	Ex	17.32 ± 1.78	23.16 ± 2.35	28.53 ± 1.83	31.44 ± 1.91	32.83 ± 1.93
	Effect	s ^#^	s ^#^	s ^#^	s ^#^	s ^#^

* Standard deviation. ^#^ Statistically significant difference between Ex and De. Ex—expected; De—designated; add—an additive effect: expected and designated values reveal differences lower than 5%. s—a synergistic effect: designated values are more than 5% lower for AAEAC when compared with expected values. an—an antagonistic effect: designated values are more than 5% higher for AAEAC when compared with expected values [43].

**Table 10:** The value of ΔA of Qui1–Qui3 ([Qui1] = [Qui2] = [Qui3] = 5 × 10^−4^ M), HSA ([HSA] = 2.5 × 10^−4^ M), and (Qui1 + HSA)_complex_, (Qui2 + HSA)_complex_, (Qui3 + HSA)_complex_ ([Qui] = 5 × 10^−4^ M; [HSA] = 2.5 × 10^−4^ M; [Qui]:[HSA] 2:1 molar ratio); FRAP assay.

FRAP Assay ΔA (Mean ± SD *)					
Samples	Time [min]				
	15	30	45	60	90
HSA	ND **	ND **	ND **	ND **	ND **
Qui1	0.02 ± 0.00	0.02 ± 0.00	0.02 ± 0.01 ^b^	0.03 ± 0.01	0.04 ± 0.01
Qui2	0.07 ± 0.00	0.08 ± 0.01 ^c^	0.08 ± 0.00 ^c^	0.09 ± 0.00 ^c^	0.09 ± 0.01
Qui3	0.39 ± 0.00 ^d^	0.43 ± 0.01	0.45 ± 0.02 ^d^	0.48 ± 0.02 ^d^	0.50 ± 0.02 ^d^
(Qui1 + HSA)_complex_	ND **	ND **	0.04 ± 0.01 ^b^	0.01 ± 0.01 ^a^	0.06 ± 0.01
(Qui2 + HSA)_complex_	ND **	0.01 ± 0.00 ^c^	0.06 ± 0.01 ^c^	0.06 ± 0.01 ^c^	0.09 ± 0.01
(Qui3 + HSA)_complex_	0.32 ± 0.01 ^d^	0.43 ± 0.04	0.56 ± 0.05 ^d^	0.57 ± 0.06 ^d^	0.72 ± 0.09 ^d^

* Standard deviation. ** ND—No data; indeterminate values due to too low reduction potential of samples in analysed conditions. ^a^ the value of ΔA without statistically significant difference from zero. ^b^ statistically significant difference between the value of ΔA in the case of Qui1 and Qui1-HSA_complex._^c^ statistically significant difference between the value of ΔA in the case of Qui2 and Qui2-HSA_complex._^d^ statistically significant difference between the value of ΔA in the case of Qui3 and Qui3-HSA_complex._

**Table 11:** Expected versus designated values of antioxidant activity AAERP of the tested samples; FRAP assay.

FRAP Assay AAERP (Mean ± SD *) [µM AA]					
Samples	Time [min]				
	15	30	45	60	90
HSA	ND **	ND **	ND **	ND **	ND **
Qui1	15.79 ± 0.74	10.38 ± 0.27	13.28 ± 1.74 ^b^	13.57 ± 1.35 ^b^	13.76 ± 1.08 ^b^
Qui2	26.98 ± 0.10	20.61 ± 1.23	24.37 ± 0.63	21.32 ± 0.22	20.63 ± 1.37
Qui3	89.45 ± 1.03	78.56 ± 1.64	85.42 ± 3.40	77.50 ± 2.64	79.24 ± 2.71
(Qui1 + HSA)_complex_	ND **	ND **	9.13 ± 1.84 ^b^	7.68 ± 1.57 ^b^	9.63 ± 1.36 ^b^
(Qui2 + HSA)_complex_	ND **	10.29 ± 0.62 ^c^	12.08 ± 0.85 ^c^	14.08 ± 0.72 ^c^	14.29 ± 1.36 ^c^
(Qui3 + HSA)_complex_	80.79 ± 2.82 ^d^	81.11 ± 6.50	95.57 ± 8.50 ^d^	88.10 ± 7.98 ^d^	104.18 ± 12.55 ^d^

* Standard deviation. ** ND—No data; indeterminate values due to too low reduction potential of samples in analysed conditions. ^b^ statistically significant difference between the value of ΔA in the case of Qui1 and Qui1-HSA_complex._^c^ statistically significant difference between the value of ΔA in the case of Qui2 and Qui2-HSA_complex._^d^ statistically significant difference between the value of ΔA in the case of Qui3 and Qui3-HSA_complex._

**Table 12:** Results of analysis of the type of interaction effect between Qui1–Qui3 and HSA.

FRAP Assay AAERP (Mean ± SD *) [µM AA]					
Sample	Time [min]				
	15	30	45	60	90
(Qui1 + HSA)_complex_	** ND	** ND	an	an	an
(Qui2 + HSA)_complex_	** ND	an	an	an	an
(Qui3 + HSA)_complex_	an	add	s	s	s

** ND—No data. add—an additive effect: expected and designated values reveal differences lower than 5%. s—a synergistic effect: designated values are more than 5% lower for AAEAC when compared with expected values. an—an antagonistic effect: designated values are more than 5% higher for AAEAC when compared with expected values [43].

**Table 13:** Experimental dataset—calibration curves and value of R^2^ (for DPPH, ABTS, FRAP assays).

	Time [min]	Calibration Curve Patterns	R^2^
DPPH assay	5	y = 1.707x + 0.636	0.99540
	15	y = 1.635x + 3.627	0.99872
	25	y = 1.611x + 4.489	0.99984
	45	y = 1.555x + 4.813	0.99849
	60	y = 1.515x + 5.929	0.99521
ABTS assay	5	y = 1.434x + 2.341	0.99547
	15	y = 1.411x + 2.407	0.99658
	25	y = 1.373x + 2.732	0.99668
	45	y = 1.357x + 2.152	0.99663
	60	y = 1.343x + 3.451	0.99755
FRAP assay	15	y = 0.005x − 0.008	0.99152
	30	y = 0.006x + 0.016	0.99156
	45	y = 0.006x + 0.003	0.99593
	60	y = 0.007x − 0.006	0.99608
	90	y = 0.007x + 0.006	0.99463

## Data Availability

The data presented in this study are available on request from the corresponding author.

## Supplementary Materials

The following supporting information can be downloaded at: https://www.mdpi.com/article/10.3390/molecules28010320/s1. Spectroscopic data of compounds: Qi1, Qi2, Qi3.

## Sample Availability

Samples of the compounds are not available from the authors.
