# Supplementary material for: New Synthetic Quinoline (Qui) Derivatives as Novel Antioxidants and Potential HSA’s Antioxidant Activity Modulators—Spectroscopic Studies
Source: Molecules. 2022 Dec 30;28(1):320. doi: 10.3390/molecules28010320 (PMC9822009; doi:10.3390/molecules28010320)
Supplement: Supplementary file 1 [file molecules-28-00320-s001.zip › molecules-2098349-supplementary.pdf]

Supplementary Materials

New Synthetic Quinoline (Qui) Derivatives as Novel Antioxidants and Potential HSA`s Antioxidant Activity Modulators - Spectroscopic Studies

Wojciech Rogóż [1], Aleksandra Owczarzy [1], Karolina Kulig [1], Jadwiga Pożycka [1], Andrzej Zięba [2] and Małgorzata Maciążek-Jurczyk [1,*]

[1] Department of Physical Pharmacy, Faculty of Pharmaceutical Sciences in Sosnowiec, Medical University of Silesia, 40-055 Katowice, Poland

[2] Department of Organic Chemistry, Faculty of Pharmaceutical Sciences in Sosnowiec, Medical University of Silesia, 40-055 Katowice, Poland

* Correspondence: mmaciazek@sum.edu.pl; Tel.: +48-32-364-15-80

Spectroscopic data of compounds: **Qi1, Qi2, Qi3**

1-methyl-3-(allylthio)-4-(4′-methylphenylamino)quinolinium bromide (**Qi1**), ^{1}H NMR (DMSO, 600 MHz) δ(ppm): 2.35 (s, 3H, CH_3), 3.45-3.56 (d, *J*=7.8 Hz, $C\underline{H_2}$-CH), 4.28 (s, 3H, NCH_3), 4.85-4.98 (m, 2H, CH=$C\underline{H_2}$), 5.61-5.78 (m, 1H, $C\underline{H}$=CH_2), 7.08-7.18 (m, 2H, H3′,5′), 7.18-7.28 (m, 2H, H2′,6′), 7.63-7.72 (m, 1H, H6), 7.98-8.08 (m, 1H, H7), 8.14-8.18 (m, 1H, H8), 8.20-8.30 (m, 1H, H5), 9.08 (s, 1H, H2), 10.56 (s, 1H, NH); ^{13}C NMR (DMSO, 150.9 MHz) δ (ppm): 21.16 ($C\underline{C}H_3$), 38.82 (CH_2), 42.83 ($N\underline{C}H_3$), 110.38 (C3), 119.12 (CH=$\underline{C}H_2$), 119.33 (C4a), 119.41 (C8), 124.52 (C3′,5′), 125.92 (C5), 127.58 (C6), 130.20 (C2′,6′), 133.51 (CH), 134.58 (C7), 136.43 (C4′), 138.22 (C1′), 139.07 (C8a), 152.73 (C2), 156.05 (C4); ESI-HRMS, Calculated for $C_{20}H_{21}N_2S$ ($[M]^+$): 321.1425, Found: 321.1414.

1-methyl-3-(allylthio)-4-(3′-hydroxyphenylamino)quinolinium bromide (**Qi2**), ^{1}H NMR (DMSO, 600 MHz) δ(ppm): 3.53-3.59 (d, *J*=7.2Hz, $C\underline{H_2}$-CH), 4.30 (s, 3H, NCH_3), 4.92-5.04 (m, 2H, CH=$C\underline{H_2}$), 5.65-5.75 (m, 1H, $C\underline{H}$=CH_2), 6.60-6.65 (m, 1H, H6′), 6.65-6.73 (m, 1H, H2′), 6.73-6.77 (m, 1H, H4′), 7.16- 7.24 (m, 1H, H5′), 7.65-7.73 (m, 1H, H6), 8.02-8.08 (m, 1H, H7), 8.16-8.22 (m, 1H, H8), 8.22-8.28 (m, 1H, H5), 9.11 (s, 1H, H2), 9.81 (s, 1H, OH), 10.49 (s, 1H, NH); ^{13}C NMR (DMSO, 150.9 MHz) δ (ppm): 38.87 (CH_2), 42.92 (CH_3), 111.09 (C3), 111.41 (C2′), 114.19 (C6′), 114.94 (C4′), 119.12 (CH=$\underline{C}H_2$), 119.41 (C8), 119.45 (4a), 126.05 (C5), 127.63 (C6), 130.48 (C5′), 133.54 (CH), 134.62 (C7), 139.07 (C8a), 141.77 (C1′), 152.65 (C2), 155.98 (C4), 158.71 (C3′); ESI-HRMS, Calculated for $C_{19}H_{19}ON_2S$ ($[M]^+$): 323.1218, Found: 323.1223.

1-methyl-3-(allylthio)-4-(4′-hydroxyphenylamino)quinolinium bromide (**Qi3**) ^{1}H NMR (DMSO, 600 MHz) δ(ppm): 3.45-3.50 (d, *J*=7.8Hz, $C\underline{H_2}$-CH), 4.23 (s, 3H, NCH_3), 4.92-5.00 (m, 2H, CH=$C\underline{H_2}$), 5.70-5.78 (m, 1H, $C\underline{H}$=CH_2), 6.80-6.86 (m, 2H, H3′,5′), 7.06-7.13 (m, 2H, H2′,6′), 7.60-7.66 (m, 1H, H6), 7.99-8.06 (m, 1H, H7), 8.09-8.12 (m, 1H, H8), 8.12-8.17 (m, 1H, H5), 8.98 (s, 1H, C2), 9.82 (s, 1H, OH), 10.44 (s, 1H, NH); ^{13}C NMR (DMSO, 150.9 MHz) δ(ppm): 38.82 (CH_2), 42.61 (CH_3), 108.97 (C3), 116.34 (C3′,5′), 116.55 (C4′), 118.59 (CH=$\underline{C}H_2$), 119.10 (C8), 119.29 (C4a), 125.97 (C5), 126.87 (C2′,6′), 127.19 (C6), 133.53 (CH), 134.50 (C7), 139.28 (C8a), 152.60 (C2), 156.37 (C4), 157.07 (C1′); ESI-HRMS, Calculated for $C_{19}H_{19}ON_2S$ ($[M]^+$): 323.1218, Found: 323.1203.
